# Intestinal microbiota: a potential target for the treatment of postmenopausal osteoporosis

**DOI:** 10.1038/boneres.2017.46

**Published:** 2017-10-04

**Authors:** Xin Xu, Xiaoyue Jia, Longyi Mo, Chengcheng Liu, Liwei Zheng, Quan Yuan, Xuedong Zhou

**Affiliations:** 1 State Key Laboratory of Oral Diseases, West China Hospital of Stomatology, Sichuan University, Chengdu, China; 2 Department of Cariology and Endodontics, West China Hospital of Stomatology, Sichuan University, Chengdu, China; 3 Department of Periodontics, West China Hospital of Stomatology, Sichuan University, Chengdu, China; 4 Department of Pediatric Dentistry, West China Hospital of Stomatology, Sichuan University, Chengdu, China; 5 Department of Dental Implantology, West China Hospital of Stomatology, Sichuan University, Chengdu, China

## Abstract

Postmenopausal osteoporosis (PMO) is a prevalent metabolic bone disease characterized by bone loss and structural destruction, which increases the risk of fracture in postmenopausal women. Owing to the high morbidity and serious complications of PMO, many efforts have been devoted to its prophylaxis and treatment. The intestinal microbiota is the complex community of microorganisms colonizing the gastrointestinal tract. Probiotics, which are dietary or medical supplements consisting of beneficial intestinal bacteria, work in concert with endogenous intestinal microorganisms to maintain host health. Recent studies have revealed that bone loss in PMO is closely related to host immunity, which is influenced by the intestinal microbiota. The curative effects of probiotics on metabolic bone diseases have also been demonstrated. The effects of the intestinal microbiota on bone metabolism suggest a promising target for PMO management. This review seeks to summarize the critical effects of the intestinal microbiota and probiotics on PMO, with a focus on the molecular mechanisms underlying the pathogenic relationship between bacteria and host, and to define the possible treatment options.

## Introduction

Postmenopausal osteoporosis (PMO) is an estrogen deficiency-induced metabolic bone disorder characterized by reduced bone strength, which increases the risk of fracture in postmenopausal women.^1^ The onset of PMO is occult, without any obvious symptoms until a fracture occurs. The most prevalent complication is a fragility fracture, which often occurs in the hip, femur, or spine under non-traumatic or mildly traumatic conditions, resulting in pain, malformation, dysfunction, and even death. Studies showed that the mortality rate associated with a hip fracture was 17% in the first year^2^ and ~12%–20% within the two following years.^3^ PMO is also a potential risk factor for oral bone loss and aggressive periodontitis in postmenopausal females. PMO animal models showed an equivalent bone loss in alveolar bone and femurs.^4^ Compared with healthy postmenopausal women, patients afflicted with PMO also exhibited an inclination to more bone loss and lower bone mineral density (BMD) in the jaw, especially in postmenopausal females with preexisting periodontitis who suffered from accelerated alveolar bone loss under routine treatment.^5–7^ In addition to bone loss and microstructural deterioration, PMO affects the osseous formation processes. Delayed osseous maturation and reduced bone regeneration during bone healing in ovariectomized (OVX) rats were reported.^8,9^ The high morbidity and serious complications of PMO have attracted major research efforts on its prophylaxis and treatment for decades. Current medications for the treatment of PMO include bisphosphonates, raloxifene, teriparatide and calcitonin, denosumab, estrogen and menopausal hormone therapy, and so on. These medications can prevent bone loss and increase bone mineral density, with a decreased risk of fractures in the vertebra, hip, or long bones.^1,10^ All of these pharmacological agents can reduce bone resorption by inhibiting osteoclasts, except teriparatide, which acts as an anabolic agent by activating or increasing osteoblast activity and prompting bone formation.^1,11^ Recent studies have demonstrated a close relationship between the intestinal microbiota and bone metabolism,^12–15^ providing evidence that the intestinal microbiome may serve as a potential therapeutic target for the treatment of PMO.

## The intestinal microbiota and its regulators

**Figure 1:**
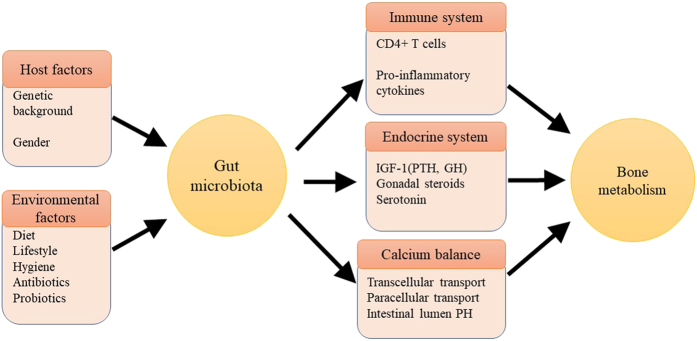
Regulators of the gut microbiota and mechanisms by which the gut microbiota regulates bone metabolism. Shaped by both host and environmental factors, the gut microbiota regulates bone metabolism through various pathways, including the immune system, endocrine system, and influences on calcium balance.

The intestinal microbiota is the collection of microorganisms that colonize the gastrointestinal tract, which consists of approximately 10 trillion bacteria.^16^ Obligate anaerobes such as Bacteroidetes and Firmicutes are the predominant residents of the healthy gastrointestinal tract, outnumbering aerobes and facultative anaerobes.^16,17^ On the basis of their roles in maintaining human health, intestinal microorganisms can be categorized into beneficial, harmful and neutral bacteria. Both host and environmental factors can shape intestinal microbial composition and structure (Figure 1). Animal experiments^18–21^ and twin studies^22,23^ revealed that the host genetic background had a significant impact on the abundance of the intestinal microbiota and the predisposition to the colonization of pathogens (for example, *Escherichia coli*). Though still disputed, gender may be another host factor affecting intestinal microbiome species diversity.^24,25^ Environmental factors, including diet, lifestyle, hygiene, antibiotic treatment, and probiotics, also contribute to the alteration of the intestinal microbiota composition.^26–31^ Notably, the effects of diet and antibiotics on the intestinal microbiota also depend on the host genetic background.^32,33^

Probiotics are defined as dietary or medical supplements consisting of live bacteria that can benefit the host if provided in adequate quantities.^34–36^ Currently, ~20 types of beneficial bacteria are used in probiotic supplements. They are generally classified into five categories, including lactobacilli, bifidobacteria, streptococci, yeast, and others.^37^ Lactobacilli and bifidobacteria are the most commonly used probiotics. Probiotics can selectively ferment prebiotics, which contain soluble dietary fibers such as oligosaccharides and inulin, facilitating the production of beneficial products conducive to the growth of certain probiotics such as bifidobacteria.^34,38,39^ However, it is still disputed whether probiotics can alter the gut microbiota composition. Randomized controlled trials (RCTs) in healthy adults indicated that probiotic intervention or probiotics-fermented products resulted in changes in intestinal microbiota composition or diversity.^40–43^ Although probiotics promoted the significant increase of certain bacteria, *Bacteroides* was the dominant genus under probiotics administration, while other bacteria such as Clostridiales were inhibited.^40,41^ In addition, the effect of probiotics on Clostridiales genera may be associated with the initial status of the intestinal microbiome and butyrate concentrations.^41^ RCTs in elder adults showed that the age-associated intestinal microbiota imbalance was restored by probiotic-based functional foods, with increased resident probiotic-related bacteria and decreased emergence of opportunistic pathogens.^44,45^ Animal experimentation also showed that probiotic administration improved the intestinal microbiota composition in hyperlipidemic rats by recovering the abundance of *Bacteroidetes* and *Verrucomicrobia* and reducing *Firmicutes*.^46^ However, another RCT in healthy adults demonstrated that *Lactobacillus rhamnosus GG* (LGG) supplement induced no alteration in gut microbiota composition or diversity stability, except for a transient increased fecal excretion of probiotic-associated bacteria during the intervention.^47^ In addition, one RCT in healthy subjects and patients with irritable bowel syndrome (IBS) showed parallel, transient, and distinct increases in probiotics but limited changes in other specific bacteria in fecal samples of both healthy and IBS-afflicted subjects with *Bifidobacterium infantis* intervention.^48^

## The intestinal microbiota regulates bone metabolism

### Involvement of the intestinal microbiota in bone metabolism

The dynamic homeostasis of the gut microbiome is critical to health. Accumulating evidence has demonstrated that the gut microbiota is associated with physiological bone metabolism and a range of inflammatory or metabolic bone diseases.^12–15,49,50^ In animal experimentation, germ-free mice showed higher trabecular volume bone mineral density (vBMD) and improved histomorphologic indices in trabecula compared with conventionally raised (CONV-R) mice.^12^ However, both trabecular BMD and cortical cross-sectional area decreased when germ-free mice were recolonized by the gut microbiota, indicating that the gut microbiota is a major regulator of bone mass.^12^ Microbial recolonization in germ-free mice induced an incipient acute decrease in bone mass but predominantly led to bone formation with a longer duration, leading to a new equilibrium in bone mass.^14^ Furthermore, germ-free mice colonized with immature gut microbiota from donors of different ages or nutritional statuses showed varied femoral phenotypes, suggesting that the impact of the gut microbiota on bone morphologic properties is age/nutrition dependent.^13^ Compromised bone biomechanical properties in mice was also induced by an altered gut microbiota resulting from immunodeficiency or long-term antibiotic intervention during growth.^15^ In addition, through post-weaning exposure to low-dose penicillin (LDP) or by introducing LDP to their mother in pregnancy, adult offspring with a perturbed gut microbiota showed altered bone mineral content (BMC) and BMD.^51^ In addition to physiological condition, inflammatory, or metabolic bone diseases, such as metabolic osteoarthritis, osteoporosis, autoinflammatory osteomyelitis, are also associated with gut microbial alteration.^49,50,52,53^ The abundance of gut bacteria *Lactobacillus spp.* and *Methanobrevibacter spp.* was shown to have a significant relationship with the prediction of osteoarthritis assessed by the Modified Mankin Score in rats.^50^ Gut microbiota modified by diet regulated the production of IL-1*β* (Interleukin-1beta) and prevented the spontaneous development of osteomyelitis in Pstpip2cmo mice predisposed to autoinflammatory osteomyelitis.^52,53^

### Mechanisms by which the gut microbiota regulates bone metabolism

Gut microbiota can regulate bone metabolism, but the exact mechanisms are still unclear. Multiple approaches through which gut microbiota may regulate bone metabolism have been proposed, including actions on the immune system, endocrine system, and calcium absorption (Figure 1).

#### (a) The gut microbiota regulates bone metabolism through the immune system

Recent studies have revealed a close interrelationship between the immune system and bone metabolism, leading to the development of “osteoimmunology,” which highlights the role of immune-related factors in modulating bone remodeling.^54,55^ In immune-mediated bone metabolism, the RANKL (receptor activator NF kappa B ligand)-RANK-OPG axis and immunoreceptor tyrosine-based activation motif (ITAM) pathway play key roles in physiological bone turnover and bone diseases.^54,56^ Recently, it has been widely recognized that the gut microbiota can interact with the host immune system and further influence host health.^57–59^ One study showed that altered immune status in germ-free mice (for example, decreased pro-inflammatory cytokines, fewer CD4^+^ T cells and reduced osteoclast/precursor cells in bone marrow) may account for the higher bone mass than in CONV-R mice.^12^ Intestinal segmented filamentous bacteria in mice were shown to promote the production of IL-17 and IFN-*γ* (Interferon-gamma), both of which played critical roles in the formation of osteoclasts and osteoblasts.^60–62^ These studies suggest that the gut microbiota regulates bone metabolism by altering host immune status.

#### (b) The gut microbiota regulates bone metabolism through the endocrine system

In addition to the immune system, hormones are regarded as another important regulator of bone metabolism. As an autocrine or paracrine growth factor, insulin-like growth factor-1 (IGF-1) can promote the differentiation and growth of bone cells, including osteoblasts, osteoclasts, and chondrocytes, and enhance normal interactions among them.^63–65^ Moreover, the IGF-1 signaling pathway is involved in the regulation of bone metabolism via both growth hormone and parathormone.^64^ Intermittent administration of parathormone promoted bone formation by increasing local IGF-1 production and activating the IGF-1 signaling pathway in bone.^64^ Growth hormone can directly or IGF-1-dependently target the growth plate to promote cartilage formation and longitudinal bone growth.^66,67^ Moreover, gonadal steroids, including estrogen and androgen, play key roles in the regulation of bone mass and turnover in bone metabolism.^68–70^ Furthermore, serum neurotransmitter 5-hydroxytryptamine, namely, circulating serotonin with a hormone-like effect, can stimulate or inhibit bone formation, and dual-directional effects may be gender/age dependent.^71–74^ The gut microbiota, which is currently considered a novel “endocrine organ” of the human body, can engage in an interplay with the endocrine system (for example, hypothalamic–pituitary–adrenal axis) and secrete hormones or hormone-like products to regulate host hormone levels, further influencing host health status.^75,76^ In animal experimentation, gut microbial colonization in germ-free mice significantly increased the serum IGF-1 level, resulting in bone growth and normalized bone mass.^14^ Isoflavones, the compounds classified as phytoestrogens and structurally similar to endogenous estrogen, were converted into more the estrogenic metabolite equol by specific gut microorganisms such as rod-shaped and gram-positive anaerobic bacteria in ~30%–50% of humans.^77–81^ Polycyclic aromatic hydrocarbons—contaminants widely present in nature—can be bio-transformed into products with estrogenic activity by the human colon microbiota.^82^ A recent study showed that the gut microbiota, especially spore-forming bacteria, can enhance the biosynthesis of serotonin by colonic enterochromaffin cells.^83^ Despite the lack of direct evidence, it has been suggested that gut microbiota-bone communication likely depends on the endocrine system or hormone-like substances.

#### (c) The gut microbiota regulates bone metabolism by influencing calcium absorption

Gut microbiota can affect the absorption of skeletal development-related nutrients such as calcium and vitamin D. Calcium, the dominant mineral component in bone, is essential for bone health. Calcium absorption can be facilitated by vitamin D. Either dietary calcium deprivation or vitamin D deficiency may induce osteoporosis.^84^ Sufficient calcium consumption can be a prophylactic measure against osteoporosis and relevant fracture.^85^ A clinical study in adolescent girls showed decreased bone resorption in the presence of high calcium consumption (47.4 mmol per day compared to the recommended 22.5 mmol per day).^86^ Some studies showed that calcium metabolism differences among ethnic groups—in terms of dietary calcium intake, renal calcium excretion, and relevant regulatory hormone or factor—were associated with bone parameters related to osteoporosis/fracture risk.^87^ In animal models, a low-calcium diet alone can lead to bone resorption, high bone turnover, and impaired bone trabecular microarchitecture in multiple bones, including the hard palate, mandible, vertebrae, femur, and proximal tibia.^88–91^

Calcium is absorbed by the active transcellular pathway (ion pumps) or passive paracellular diffusion (ion channels), depending on the level of 1,25-(OH)_2_D (1,25-dihydroxy vitamin D).^92^ The proteins involved in the transcellular pathway consist of transient receptor potential vanilloid type 6 (TRPV6/CaT1/ECaC2), which absorbs calcium from the gut lumen into cells; calbindin-D9k, which is responsible for intracellular calcium transportation; and plasma membrane calcium ATPase 1b (PMCA1b), which excretes calcium outside cells into the blood.^93^ Passive paracellular calcium diffusion occurs as calcium (Ca^2+^) flux across the intestinal epithelium and is based on tight junction (TJ) proteins between intestinal epithelial cells.^94^ Normal calcium intake rates in adults are ~30%–35%;^95,96^ these levels can be increased by probiotics, prebiotics, and synbiotics consisting of probiotics and their favorable prebiotics.^97^ Specific probiotic bacteria, such as *Lactobacillus salivarius* rather than *Bifidobacterium infantis*, stimulated calcium uptake by enterocytes in a Caco-2 cell culture model.^98^ Oligosaccharides (NDO), the dietary prebiotics containing fructooligosaccharides (FOS) and inulin, significantly facilitated intestinal calcium absorption and increased skeletal calcium content in growing and adult rats.^99–102^ Prebiotic inulin produced an enhancement in calcium absorption compared to other oligosaccharides,^99,100^ while the combination of both may act synergistically.^101,102^ In addition, a study in healthy adolescent girls demonstrated that daily administration of GOS can increase calcium absorption.^103^ Another clinical study reported the improvement of calcium absorption in young healthy women with long-term treatment with lactosucrose.^104^

As the fermentation substrates of gut microbiota, prebiotics affect bone metabolism by producing a variety of beneficial metabolites, such as short-chain fatty acids (SCFA). The potential mechanism by which SCFA regulate bone metabolism involves direct effects on proteins associated with calcium absorption. Experiments both *in vitro* and *in vivo* using animal models showed that an SCFA supplement could increase the transcriptional levels of TRPV6 and calbindin-D9k rather than PMCA1b in cultured Caco-2 human colonic epithelium and rat colorectal mucosa.^105,106^ The *TRPV6* gene was shown to contain a segment characterized by a positive response to SCFA.^105^ In addition, the response of calbindin-D9k to SCFA varied with time and SCFA dose.^106^ The upregulation of calbindin-D9k by prebiotic diet specifically occurred in the colorectal segment regardless of dietary calcium uptake and serum 1,25-(OH)_2_D level, and it was related to the transcription factors vitamin D receptor (VDR) and cdx-2.^107–109^ The SCFA butyrate resulting from the prebiotic diet can upregulate VDR, activate the cdx-2 promoter, and facilitate cdx-2 mRNA expression.^110^ Although direct evidence for the SCFA-related effect on intestinal paracellular calcium absorption is still absent, a ruminant model in which more than 50% of calcium absorption pre-intestinally occurs in the rumen manifested a dose-dependent promotion by SCFA on the ruminal calcium ion flux rate from mucosa to serum in the paracellular pathway.^111,112^ As stated above, both probiotics and prebiotics can influence intestinal epithelial permeability by regulating TJ protein expression and distribution, which possibly underlies the mechanism of prebiotic effects on paracellular calcium transport. In addition to direct action on the cellular structure involved in the calcium absorption process, prebiotics can also alter the intestinal microenvironment, thereby indirectly modulating bone metabolism. SCFA generated from prebiotics could lower the intestinal lumen pH and consequently inhibit the formation of calcium complexes, such as calcium phosphates, leading to increased calcium absorption.^113^

## Relationship between the intestinal microbiota and PMO

### PMO animal models

Current data on the relationship between intestinal microbiota and PMO are primarily obtained from animal models. The most commonly used PMO animal models are rodents submitted to either surgery or medication. Ovariectomy is the most frequently used surgery to generate PMO rodent models. Bilateral ovariectomy is used to successfully set up morbid states of PMO in the proximal tibia, distal femur and lumbar vertebra according to the guidelines for the preclinical and clinical evaluation of PMO medication issued by the United States Food and Drug Administration (FDA).^114^ Gonadotropin-releasing hormone (GnRH) agonists are frequently used to induce PMO in rodents. The long-term or high dose administration of GnRH agonists to rats typically housed under germ-free conditions ^49^ inhibits the secretion of endogenous GnRH, gonadotrophin and estrogen.^115,116^ GnRH agonist-induced bone loss is reversible. Kurabayashi *et al* found that Sprague-Dawley (SD) rats submitted to long-term GnRH agonist treatment exhibited decreased bone mass, bone density, and bone turnover that could be partially recovered after treatment interruption.^115^ Estrogen deficiency induced by either ovariectomy or GnRH agonist in murine models evidently increases bone turnover and bone loss and reduces bone mineral density and bone volume in lumbar vertebrae and long bones, thus recapitulating conditions in patients with PMO.^114,115,117^

Animal age can affect the final experimental results, as preadolescent mice undergo rapid bone growth and high bone turnover due to the presence of growth hormones.^118^ In addition, mice are likely to undergo irreversible aging symptoms^119^ and potentially develop senile osteoporosis as early as 5–6 months old. Therefore, 8- to 20-week-old rats or mice are usually used to establish PMO animal models.^49,115,118,120–128^

### PMO development depends on the intestinal microbiota and host genetic background

The intestinal microbiota is indispensable to PMO development. Compared to conventionally raised (Con-R) mice, germ-free (GF) mice showed no significant alteration in either pro-inflammatory cytokines in bone marrow or femoral trabecular parameters after PMO model establishment by the administration of GnRH agonists.^49^ However, similar to Con-R mice, GF mice colonized with a normal gut microbiota exhibited increased pro-inflammatory cytokines and impaired bone properties due to estrogen deficiency.^49^ Accordingly, intestinal microorganisms are involved in estrogen deficiency-associated trabecular bone resorption. These microorganisms may be correlated with certain trabecular bone parameters. In particular, trabecular number (Tb.N) and trabecular spacing (Tb.Sp) are influenced by the intestinal microbiota, whereas trabecular thickness (Tb.Th) is not.^49^

**Figure 2:**
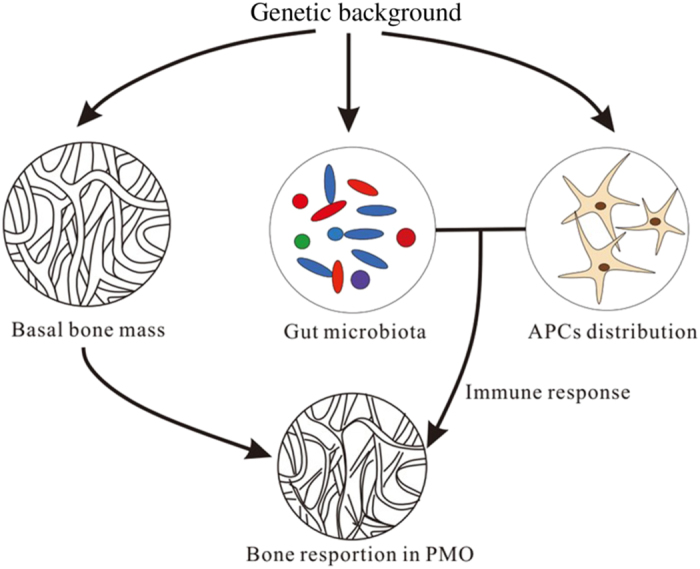
Genetic background acts on PMO bone loss. Genetic regulation affects bone loss in PMO by shaping the gut microbiota and determining basal bone mass as well as the distribution of APCs.

Bone resorption in PMO has also been shown to be closely related to genetic background (Figure 2). Previous studies have shown that estrogen deficiency-induced bone loss varies remarkably among different mouse strains.^124,126,127^ Genetic regulation can act on PMO bone loss through multiple mechanisms. Genetic background determines basal bone mass^1,122^ and the specific distribution of intestinal antigen-presenting cells (APCs) with different functions.^129^ Intestinal APCs, especially dendritic cells (DCs), present pathogenic antigens from the gut microbiota and activate CD4^+^ T cells to produce pro-inflammatory cytokines such as tumor necrosis factor-*α* (TNF-*α*), which stimulates osteoclastogenesis and induces bone loss.^130,131^ In addition, host genetic background can shape the intestinal microbiota,^20,22,23,33,132,133^ which can influence the development and activity of host immune systems ^59,134^ and thus may indirectly regulate bone loss in PMO.

### Probiotics prevent bone loss in PMO murine models

Bone loss in PMO murine models can be prevented by probiotics. Several studies have shown that bone resorption of femur and vertebra in OVX mice could be completely inhibited by the administration of probiotics such as *Lactobacillus reuteri*, LGG and the commercial mixture VSL#3.^49,118^ In addition, probiotics such as *Bifidobacterium longum*, *Lactobacillus paracasei* and a mixture of *Lactobacillus paracasei* and *Lactobacillus plantarum* alleviated femoral bone loss and increased bone mineral density in OVX rats or mice.^120,121^ Furthermore, soy skim milk fermented by *Lactobacillus paracasei subsp. paracasei NTU 101* (NTU 101F) and *Lactobacillus plantarum NTU 102* (NTU 102F) mitigated bone loss and improved the trabecular microarchitecture in OVX mice.^125^

The effects of probiotics on bone tissues depend on the systemic conditions of the host. McCabe LR *et al*^123^ showed that *L. reuteri* increased trabecular bone parameters of the femur and vertebra in healthy male mice (but not intact female mice), suggesting that estrogen level might affect the sensitivity of bone formation to *L. reuteri* in mice. *L. reuteri* may affect bone metabolism by activating the estrogen signaling pathway in male mice, whereas healthy adult female mice are impervious to *L. reuteri* due to sufficient estrogen. Notably, probiotics enhanced the trabecular bone parameters in intact female mice under inflammatory conditions after surgery.^49,135^ These results indicate that inflammatory pathways may be potential targets of probiotics to normalize bone homeostasis.

## Host and microbiota interactions in the pathogenesis and treatment of PMO

Immune responses mediated by antigens from the intestinal microbiota play a central role in the pathogenesis of PMO. Under healthy conditions, interplays between the intestinal microbiota, the intestinal epithelial barrier, and the host immune system maintain homeostasis, inhibiting the number of intestinal pathogens and maintaining musculoskeletal balance. If homeostasis is disturbed, intestinal pathogens intrude into the host through the epithelial barrier and provoke an immune response, ultimately promoting osteoclastic bone resorption and continual bone loss in PMO. Accordingly, probiotics ameliorate bone resorption and destruction by suppressing immune responses and restoring equilibrium between the intestinal microbiota and the host.

### Intestinal microbial diversity in PMO is regulated by estrogen and probiotics

**Figure 3:**
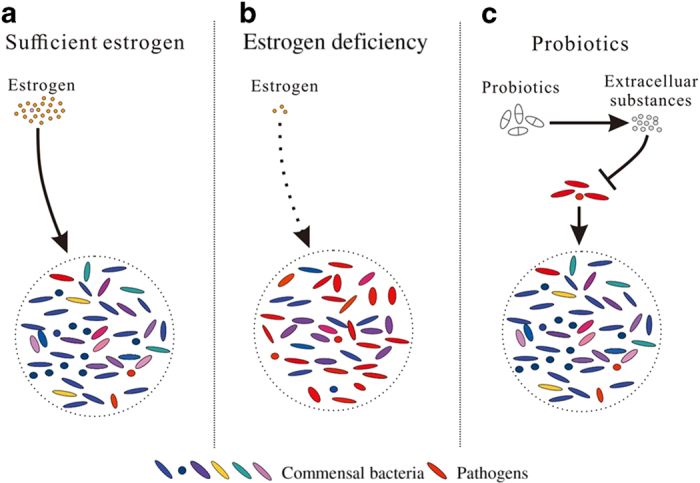
Intestinal microbial diversity in PMO is regulated by estrogen and probiotics. Healthy status can maintain gut microbial diversity and beneficial bacteria, which can activate Tregs to sustain immune homeostasis that is resistant to pathogens (**a**). Estrogen deficiency reduces gut microbial diversity and beneficial bacteria, while increased pathogens induce inflammation (**b**). Probiotics can prevent pathogens and increase gut microbial diversity by producing extracellular substances (**c**).

A healthy state and sufficient estrogen levels maintain intestinal microbial diversity (Figure 3a). Under these conditions, beneficial bacteria are predominant and stunt the growth of pathogenic species, preserving the stability of the intestinal microbiota composition. In postmenopausal women, the absence of estrogen alters intestinal microbial composition and structure, leading to decreased microbial diversity (Figure 3b). Clinical surveys of males and postmenopausal females have shown significant correlations between biodiversity (or *Clostridium* abundance) in feces and urinary levels of estrogen (or estrogen metabolites).^136,137^ Estrogen deficiency destroys intestinal microbial diversity, which is reflected as a reduction in Firmicutes populations, including *Clostridium* species.^136–138^ Firmicutes bacteria, especially *Clostridium* species, possess immune-regulatory effects that boost the formation of regulatory T cells (Tregs) and enhance their function, sustaining immune homeostasis.^139,140^ Hence, estrogen deficiency undermines intestinal microbial diversity and reduces the abundance of intestinal bacteria that are conducive to immune homeostasis, consequently facilitating pathogen reproduction and initiating an immune response.

When used to treat PMO, probiotics improve intestinal microbial constitution and restore biodiversity. Probiotics halt pathogen growth and increase intestinal microbial diversity by synthesizing extracellular compounds (Figure 3c). A study by Preidis GA *et al*^141^ showed that *L. reuteri* increased microbial diversity and homogeneity in the feces of mice by producing reuterin. Reuterin, an antibiotic compound, promotes oxidative stress in cells by inducing the modification of thiols on proteins or small molecules, which in turn suppress the growth of pathogens such as *Bacteroides* while increasing the presence of *Clostridium* species.^118,142^ Additionally, the *Lactococcus lactis* strain G50 prevented H_2_S-producing bacteria from growing, while strain H61 had an inhibitory effect on *Staphylococcus* in a mouse model of senile osteoporosis.^119,143^ However, it has not yet been demonstrated whether *L. lactis* has an equivalent role in PMO.

### Intestinal epithelial barrier function in PMO is regulated by estrogen and probiotics

The intestinal epithelium is the first barrier to physically resist intestinal pathogens. This barrier not only absorbs water and nutrients but also limits the penetration of intestinal antigens. The ability of the barrier to function properly depends on transcellular and paracellular pathways. The fundamental paracellular pathway structure is the TJ, the integrity and selective permeability of which are of vital importance to intestinal epithelial barrier function. TJs are protein complexes consisting of claudin, occludin, and zonula occludens (ZO) proteins, which together allow selective passage of ions and small molecules.^144–150^ TJ permeability can be represented by transepithelial electrical resistance (TER); higher TER usually indicates lower permeability.^151,152^ Both physiological and pathological stimuli can affect the production and distribution of TJ proteins, thereby modulating intestinal epithelial permeability. TJ proteins are mainly regulated by phosphorylation through protein kinase A (PKA), protein kinase C (PKC), protein kinase G (PKG), serine/threonine (Ser/Thr) kinases, Rho, mitogen-activated protein kinase (MAPK), phosphatidylinositol-3-kinase/Akt (PI3K/Akt), and myosin light chain kinase (MLCK).^144,150^

**Figure 4:**
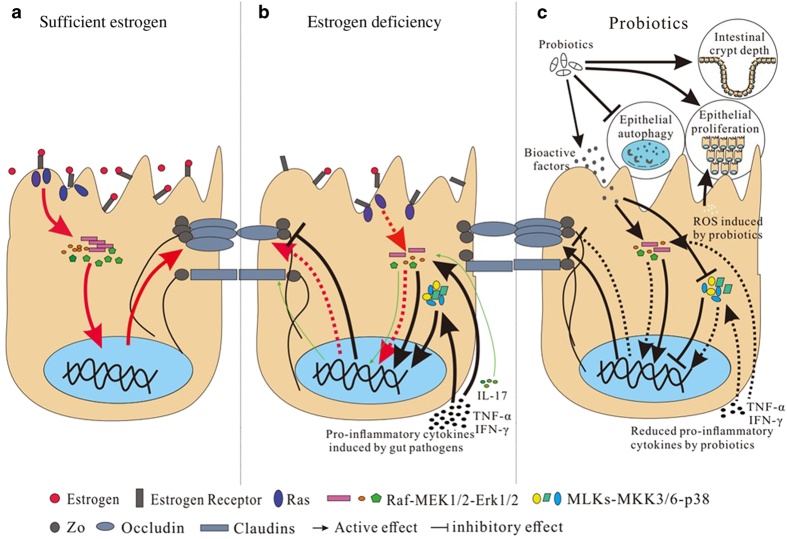
Intestinal epithelial barrier function in PMO is regulated by estrogen and probiotics. Sufficient estrogen can prompt the expression of tight junction (TJ) proteins through the Raf-MEK1/2-ERK1/2 pathway to enhance the gut epithelial barrier (**a**), while this active effect on TJ is weakened by estrogen deficiency (**b**). Under estrogen deficiency, pathogen-induced pro-inflammatory cytokines such as TNF-*α* and IFN-*γ* reduce the production of TJ proteins through both the Raf-MEK1/2-ERK1/2 and MLKs-MKK3/6-p38 pathways and compromise the gut epithelial barrier (**b**). The positive action of IL-17 on TJ proteins (thin green arrows in **b**) fails to completely compensate for the adverse effect of TNF-α and IFN-γ. Probiotics can enhance the gut epithelial barrier by regulating the production and distribution of TJ proteins and affecting the growth and movement of intestinal epithelial cells (**c**).

Sufficient levels of estrogen activate the GTP-binding protein Ras and a series of kinases present in cytoplasm (Raf, MEK1/2, and ERK1/2) through estrogen receptors on the intestinal epithelium; they also maintain relatively high levels of occludin protein expression (Figure 4a).^144,153–155^ As a result of this paracellular pathway, the intestinal epithelial barrier exhibits increased TER and can prevent pathogen invasion. Estrogen deficiency weakens the effect of the aforementioned estrogen-associated pathway, leading to increased intestinal epithelial permeability.^156^ Antigens from intestinal pathogens initiate inflammatory cascades across the epithelial barrier, leading to the production of pro-inflammatory cytokines such as tumor necrosis factor-α (TNF-α) and interferon-γ (IFN-γ). TNF-α and IFN-γ downregulate the TJ proteins occludin and ZO-1 via Raf-MEK1/2-ERK1/2 or MLKs-MKK3/6-p38 in the MAPK pathway and further compromise the intestinal epithelial barrier.^157^ In addition, the pro-inflammatory factor interleukin-17 (IL-17) can increase claudin-1 protein expression and reinforce the intestinal epithelial barrier through Ras-Raf-MEK1/2-ERK1/2 in the MAPK pathway.^158^ However, the positive action of IL-17 fails to completely compensate for the adverse effect of TNF-α and IFN-γ because TNF-α and IFN-γ may be central players in the immune responses elicited by intestinal bacteria. Hence, estrogen deficiency increases intestinal epithelial permeability (Figure 4b), facilitating the intrusion of intestinal pathogens and provoking immune reactions, and ultimately resulting in increased osteoclastic bone resorption and continual bone loss in PMO.

When used to treat PMO, probiotics fortify the intestinal epithelial barrier to protect the host against intestinal pathogen invasion (Figure 4c). Probiotics regulate the production and distribution of TJ proteins and reduce intestinal epithelial permeability by inducing changes in TJ-related gene expression. *In vitro* experiments have confirmed that *L. plantarum* can promote the production and rearrangement of claudin-1, occludin and ZO-1 proteins in the Caco-2 human colon adenocarcinoma cell line in a dose-dependent manner.^159,160^ *Bifidobacteria infantis* was found to increase ZO-1 and occludin protein expression by inhibiting pro-inflammatory cytokines or through the secretion of polypeptide bioactive factors to augment Erk levels while decreasing p38 levels.^161^ The probiotic mixture VSL#3 also promoted the expression and redistribution of occludin, ZO-1, and claudin-1 proteins in a mouse model of acute colitis.^162^ The potential mechanism for the probiotic regulation of TJ proteins probably involves SCFAs as fermentation products, especially butyrate, which could stimulate the reorganization of TJ proteins and promote TJ assembly by up-regulating AMP-activated protein kinase (AMPK) activity in the Caco-2 cell model, resulting in increased TER and an enhanced intestinal epithelial barrier.^163^ In addition, probiotics affected the growth and movement of intestinal epithelial cells by altering gene expression related to protein synthesis, metabolism, cell adhesion and apoptosis.^162,164^ *L. reuteri* substantially promoted intestinal epithelial cell migration and proliferation and increased intestinal crypt depth, ultimately improving the absorptive function of the intestinal epithelial barrier.^141^ Both LGG and *L. plantarum* can stimulate the intestinal epithelium to produce physiological levels of reactive oxygen species (ROS), which act as a second messenger to activate the Erk/MAPK pathway and consequently lead to intestinal epithelial proliferation.^165,166^ Probiotics also offer resistance against the toxic effects produced by intestinal pathogens on the intestinal epithelium. Bifidobacteria reduce the production of autophagy-related proteins and further prevent intestinal epithelial autophagy triggered by endotoxins from gram-negative bacteria.^167^

### Host immune responses in PMO are regulated by estrogen and the intestinal microbiota

The immune system is the final barrier to intestinal pathogen invasion and is also a critical target for PMO treatment. APCs in the intestinal lamina propria can be divided into dendritic cells (DCs) and macrophages.^129^ Although all macrophages and DCs can induce Foxp3^+^ Treg cell differentiation, macrophages with a higher T cell/APC ratio are more efficient than DCs.^129^ By contrast, DCs only partially induce Th17 cell differentiation.^129^ Treg cells are a subset of immunocytes with inhibitory effects on the differentiation and function of Th1, Th2, and Th17 cells.^130^ In addition, Treg cells can inhibit osteoclast formation by cell-to-cell contact via the cytotoxic T lymphocyte antigen (CTLA-4) or by secreting anti-inflammatory cytokines such as IL-4, IL-10, and transforming growth factor-β (TGF-β).^168–171^ Th17 cells, a subgroup of T cells, stimulate osteoclast formation and bone resorption by producing high levels of IL-17, RANKL, and TNF-*α*.^172^

**Figure 5:**
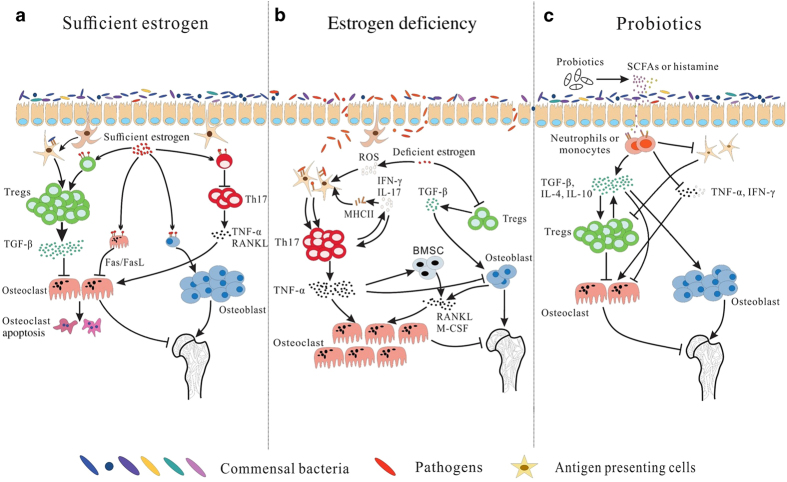
Host immune responses in PMO are regulated by estrogen and intestinal microbiota. Both beneficial gut bacteria and sufficient estrogen activate Tregs, which produce TGF-β to prevent osteoclastogenesis and induce osteoclast apoptosis; estrogen prompts osteoblast formation to improve bone mass and structure (**a**). Estrogen deficiency reduces osteoblast formation; the invasion of pathogens activates CD4+T cells including TH17, which mainly produce TNF-α to promote osteoclastogenesis, leading to bone loss and microstructural destruction (**b**). Probiotics can regulate immune responses by secreting small molecules such as SCFAs and histamine (**c**).

Both adequate estrogen levels and intestinal microbial diversity are needed to maintain immune homeostasis (Figure 5a). *Clostridium* improves the aggregation, quantity and function of Treg cells to create an environment abundant in TGF-β, which consequently prevents osteoclastogenesis.^139^ Estrogen protects bone by down-regulating immune responses and modulating osteoblast/osteoclast equilibrium.^173^ Estrogen not only activates the apoptosis-promoting Fas/FasL pathway through direct interaction with osteoclasts ^174–177^ but also indirectly increases TGF-β production by Treg cells and decreases the production of TNF-a and RANKL by Th17 cells, ultimately promoting osteoclast apoptosis.^131,168,169,171,178,179^ Furthermore, estrogen exerts anti-apoptotic effects on osteoblasts and osteocytes through the ERK pathway.^177,180^

Estrogen deficiency and reduced intestinal biodiversity have negative effects on bone (Figure 5b). Pathogenic antigens cross the intestinal epithelium and trigger inflammatory immune responses that are mainly mediated by T cells. Estrogen deficiency boosts the antigen presentation of DCs and macrophages through multiple pathways. Upon estrogen depletion, ROS excessively accumulate in bone marrow cells.^181,182^ ROS enhance the antigen-presenting function of DCs, which further activates CD4^+^T cells to produce IFN-γ. The enhanced production of IFN-γ in turn improves the antigen-presenting ability of bone marrow macrophages (BMM) by up-regulating MHC II molecules.^183–187^ In addition, estrogen deficiency upregulates co-stimulator CD80 to activate bone marrow DCs.^184^ Increased antigen presentation motivates CD4^+^ cells, including IL-17-producing Th17 cells, to mediate osteoclast formation and bone resorption.^130,188^ In addition to antigen-dependent activation, increased levels of IFN-γ and IL-7, in combination with low levels of TGF-β, indirectly activate T cells in bone marrow.^130,188,189^ Activated T cells generate a considerable quantity of TNF-α, which acts as a key pathogenic factor in PMO development.^131,190–193^ TNF-α stimulates the production of RANKL and macrophage colony stimulatory factor (M-CSF); it also suppresses the production of osteoprotegerin (OPG) by inducing the expression of CD40L and the bone mass regulatory factor DLK1/FA-1.^130,194,195^ In addition, TNF-*α* acts either directly on osteoclast precursors to promote their maturation^196^ or indirectly on TNF-*α* receptor p55 to augment M-CSF- and RANKL-induced osteoclastogenesis.^131^ Furthermore, estrogen deficiency increases levels of Act1 adaptor protein on the surfaces of osteoblasts and subsequently activates the IL-17 signal pathway to promote bone resorption.^197,198^ These findings provide evidence that CD4^+^ T cells (including Th17 cells) and the pro-inflammatory cytokine TNF-*α* are primary factors responsible for bone loss mediated by intestinal bacteria in PMO.

When used for PMO treatment, probiotics also suppress bone resorption by regulating immune responses to intestinal microorganisms. Probiotics secrete small molecules to regulate the host immune response (Figure 5c). Probiotics also produce SCFAs by utilizing prebiotics.^30,34,199,200^ SCFA receptors contain GPR41 and GPR43, the latter of which is mainly found in immunocytes such as neutrophils and monocytes.^201^ SCFAs, especially butyric acid, interact with GPR43 to reduce levels of monocyte chemotactic protein 1 (MCP-1) and LPS-induced cytokines such as TNF-*α* and IFN-γ. They also up-regulate the expression of TGF-β1, IL-4 and IL-10, ultimately activating Treg cells.^120,201–205^ In addition, *L. reuteri* transforms dietary L-histidine to histamine, which inhibits the MEK1/2-ERK1/2 pathway via H2 receptors and further inhibits TNF-α production by monocytes.^206^ *Lactobacillus* also impedes DC activation during inflammation and promotes Treg differentiation by inducing the expression of molecular ligands with inhibitory effects on pertinent DNA motifs.^207^

### The intestinal microbiota and estrogen orchestrate calcium absorption

As described above, both calcium content and estrogen level are critical to bone metabolism. In postmenopausal-osteoporotic rats, combined deficiencies of dietary calcium and estrogen had a more adverse effect on bone mass and microstructure than either single deficiency, with more bone loss and more severely impaired bone properties.^88,90,208^ In addition, calcium balance can be regulated by estrogen. Under normal conditions, estrogen treatment can increase intestinal calcium absorption in rats.^209^ Accumulating evidence suggests that estrogen deficiency could induce impaired calcium absorption, which was improved by estrogen supplementation.^210–212^ The potential mechanisms of estrogen-associated regulation on calcium absorption are still disputed. Estrogen may indirectly promote vitamin D receptor (VDR) protein expression and enhance intestinal mucosal responsiveness to 1,25-(OH)_2_D, resulting in increased intestinal calcium absorption.^213,214^ However, estrogen deficiency-related calcium malabsorption may not depend on the serum 1,25-(OH)_2_D pathway. Estrogen reversed the reduced calcium absorption by directly interacting with estrogen receptor alpha (ER-*α*) on the intestine, up-regulating the calcium transport protein 1 (CaT 1) of the calcium influx channel without significantly altering serum 1,25-(OH)_2_D level.^212,215,216^ In addition, estrogen deficiency increased the urinary fractional excretion of calcium (FECa) in OVX rats.^120^

The imbalance in calcium metabolism induced by estrogen deficiency was also redressed by the application of probiotics and prebiotics for the treatment of PMO.^97^ Probiotic supplements completely inhibited the increase in FECa due to estrogen deficiency in OVX rats.^120^ Oligosaccharides (NDO), dietary prebiotics such as fructooligosaccharides (FOS), galactooligosaccharides (GOS), and inulin, can significantly promote intestinal calcium absorption and skeletal calcium retention in OVX rats, resulting in suppressed bone loss.^217,218^

### The gut microbiota produces estrogen-like metabolites with regulatory effects on bone metabolism

Estrogen plays a major role in promoting osteogenesis. The role of estrogen is not limited to the direct suppression of osteoclast activity and lifespan, facilitation of osteoblast lifespan and differentiation, or reduction of mature osteoblasts apoptosis to promote osteogenesis. It also inhibits the formation of both osteoblasts and osteoclasts from bone marrow precursors to prevent bone remodeling and regulate bone turnover.^69,70^ In the absence of estrogen due to ovariectomy or post-menopause, estrogen-deficient women exhibit accelerated bone loss and increased bone turnover as well as impaired bone microarchitectural and mechanical properties.^49,177,219^ Hormone replacement therapy (HRT), including supplementation with estrogen and progesterone, has been applied to postmenopausal women suffering from PMO and achieved favorable effects.^220^ Instead of estrogen supplementation, the gut microbiota may act as another “endocrine organ” and potentiate novel access to replenish estrogen by utilizing exogenous nutrients and producing more estrogenic substances.

Phytoestrogens, which are predominantly present in natural foods such as soy, are exogenous nutrients with structures and bioactivity similar to human intrinsic estrogens. Various metabolites produced from phytoestrogens by the gut microbiota, including equol, urolithins, and enterolignans, are characterized by higher bioavailability and respectively more estrogenic, antiestrogenic and antioxidant bioactivities than their precursors in phytoestrogens, such as isoflavones, ellagitannins, and lignans.^221^ Daidzein, the principle isoflavone in soy, has two metabolic patterns including equol and O-desmethylangolensin (O-DMA) production.^222^ Equol shows much more estrogenic bioactivity or effects than O-DMA for bone metabolism in PMO.^223^ Equol, which is mostly present as a glucuronide conjugate and binds to the estrogen receptor (ER), can suppress bone resorption, promote bone formation, and improve bone biomechanical and microstructural properties in subjects with PMO but has no impacts on bone in healthy early postmenopausal women.^224–229^ The potential mechanism that involves equol may prevent osteoclast formation, stimulate the proliferation and differentiation of osteoblasts, and increase osteocalcin level by ER.^223,230^ Additionally, equol can inhibit the expression of relevant inflammatory cytokines in bone marrow in a dose-dependent fashion due to estrogen deficiency or LPS from intestinal pathogens.^231–233^ Although produced by gut microbiota, equol may modify gut microbiota diversity and composition in turn.^231^ Isoflavone metabolism can promote the growth of *Clostridium* clusters XIVa and IV and suppress the genera *Bacteroides* and *Parabacteroides*.^234^ Nevertheless, equol production from dietary phytoestrogens has significant interpersonal variations, predominantly depending on gut microbial composition and potential correlations among the three groups of phytoestrogen metabolism as well as dietary components.^77,221,235,236^ At present, the key equol-producing gut bacteria have not yet been identified. Most studies target potential equol-producing bacteria by cultivation or sequence analysis of fecal samples. Two strains of *Eubacterium sp.* were isolated and considered the most likely equol-producing bacteria from pig feces.^237^ Another intestinal bacteria, *Slackia TM-30*, a rod-shaped and gram-positive anaerobe isolated from healthy human feces, also proved to be highly related to equol production.^78^ The sequence information for fecal samples in postmenopausal women with dietary isoflavone uptake indicated obviously higher proportions of *Eubacterium* and *Bifidobacterium* in equol-producing subjects than in equol non-producers.^238^ Other studies identified other bacteria that significantly increased in fecal samples of equol producers, including *Collinsella*, *Asaccharobacter*, *Dorea*, and *Finegoldia*.^234,239^ In terms of function, sulfate-reducing bacteria were suggested to be involved in equol production.^235^ In addition to specific gut bacteria, equol-producing capacity may inversely correlate with O-DMA production.^235^ In addition, daidzein bioavailability and the equol/O-DMA production ratio could be elevated by the combined administration of isoflavones and prebiotic oligosaccharides or probiotic bacteria such as *Lactobacillus casei*.^240–242^ However, another study showed that the combination of soy isoflavones and fructooligosaccharides had no synergistic effects on bone mineral density or bone mineral content but effectively improved bone microstructural properties, including trabecular number, thickness, and separation.^243^ Overall, the beneficial effects of phytoestrogen supplementation on PMO mainly depend on individual metabolisms involving both the appropriate gut microbiome and dietary composition.^244^

## Conclusion

Bone resorption in PMO is the consequence of interactions among the estrogen level, the intestinal microbiota, and the host immune system. When estrogen levels are deficient, bacteria and intestinal antigens cross the compromised intestinal epithelium barrier and initiate the immune responses associated with bone loss in PMO. Probiotics prevent bone resorption by restoring intestinal microbial diversity, enhancing the intestinal epithelial barrier, and normalizing aberrant host immune responses, as well as facilitating intestinal calcium absorption and the potential production of estrogen-like metabolites, as summarized in Table 1. Hence, the intestinal microbiota serves as a key factor in the pathogenesis of PMO and will also serve as a new target in the treatment of PMO.

The application of probiotics may be a promising adjuvant to current therapies. However, current studies on probiotics for PMO treatment are limited to animal studies. The translation from animal studies to clinical application faces many challenges, such as effective dosage and safety in humans. The safety and feasibility of probiotics application in humans have been demonstrated by clinical studies in specific groups, such as in healthy infants,^245^ preterm infants, children with intractable diarrhea,^246^ and children and adolescents undergoing HCT.^247^ However, in patients with predicted severe acute pancreatitis, significant increases in bowel ischemia and mortality were related to probiotic prophylaxis, as reported in the study by Besselink *et al*^248^ Hence, more studies are needed to validate the safety of probiotics and confirm the optimal dosage and the proper time and method of delivery for probiotics in the context of PMO treatment.

## Figures and Tables

**Table 1:** Current probiotics with beneficial effects on estrogen deficiency-induced bone loss

Probiotics	Research models	Outcomes	References
*Lactobacillus* spp.			
*L. rhamnosus* GG	C57Bl/6 OVX mice	Attenuates intestinal and BM inflammation and completely inhibits bone loss	^49^
	C57Bl/6 OVX mice	Reduces TJ destruction and gut epithelial permeability	^49^
	C57Bl/6 OVX mice	Affects enterocyte proliferation and migration	^165^
*L. reuteri*	Balb/c OVX mice and healthy C57Bl/6 male mice or intact female mice with inflammation	Suppresses inflammation and bone loss in OVX mice and increases bone parameters in healthy male mice	^118,123,135,206^
	Outbred CD1 neonatal mice	Increases enterocyte migration, proliferation, and crypt height	^141^
	Outbred CD1 neonatal mice or Balb/c OVX mice	Increases intestinal microbial diversity and evenness and inhibits growth of pathogens	^118,141,142^
*L. paracasei*	C57Bl/6 OVX mice	Decreases inflammatory cytokines and bone loss	^120^
*L. plantarum*	Murine and drosophila intestine or Caco-2 cell monolayers	Induces enterocyte proliferation and modulates cellular processes e.g., metabolism, adhesion and apoptosis	^164,166^
	Caco-2 cell monolayers	Promotes production and rearrangement of TJ proteins and enhances TJ integrity	^159,160^
*Lactococcus lactis*	SAMP6 mice	Inhibits H_2_S-producing bacteria and Staphylococcus	^119,143^
*Bifidobacterium* *spp*.			
*B. longum*	OVX SD rat	Reduces bone loss and enhances bone mineral density	^121^
*B. infantis*	IL-10-deficient mice	Induces rearrangement of TJ proteins and normalizes gut permeability	^161^
*Mixture*			
*L. paracasei* and *L. plantarum*	C57Bl/6 OVX mice	Decreases inflammatory cytokines and bone loss	^120^
VSL#3^a^	C57Bl/6 OVX mice	Attenuates intestinal and BM inflammation and completely inhibits bone loss	^49^
	C57Bl/6 OVX mice or BALB/c mice in acute colitis model	Promotes expression and redistribution of TJ proteins and reduces intestinal epithelial permeability	^49,162^

a The mixture VSL#3 contains *Bifidobacterium breve*, *Bifidobacterium longum*, *Bifidobacterium infantis*, *Lactobacillus acidophilus*, *Lactobacillus plantarum*, *Lactobacillus paracasei*, *Lactobacillus bulgaricus*, and *Streptococcus thermophiles*.
